# Mobile weight self-monitoring adherence and eating behavior changes: A secondary analysis of a 12-month RCT

**DOI:** 10.1177/20552076251395530

**Published:** 2025-12-10

**Authors:** Renata Savian Colvero de Oliveira, Sharon Nabwire, Silja Rantaiso, Heta Merikallio, Markku J Savolainen, Janne Hukkanen, Harri Oinas-Kukkonen

**Affiliations:** 1 Oulu Advanced Research on Service and Information Systems Unit, 6370 University of Oulu, Oulu, Finland; 2 Research Unit of Biomedicine and Internal Medicine, 6370 University of Oulu, Oulu, Finland; 3 Medical Research Center Oulu, Oulu University Hospital and 6370 University of Oulu, Oulu, Finland

**Keywords:** Behavior change support systems, digital health intervention, persuasive systems design, self-monitoring, eating behavior

## Abstract

**Background:**

Mobile health behavior change support systems (mHBCSS) have been shown effective in weight management by promoting self-monitoring, a persuasive strategy that enhances engagement and supports healthier behaviors. Daily weight tracking supports weight loss but may increase the risk of disordered eating, particularly in individuals with obesity.

**Objective:**

To assess the 12-month impact of weight self-monitoring on eating behaviors among individuals with obesity using a mHBCSS.

**Methods:**

We analyzed data from 98 participants in a 12-month randomized controlled trial using a mHBCSS. Participants were grouped into “continuous self-monitors” (monitored ≥6 months) and “non-continuous self-monitors.” Outcomes included binge eating scale (BES), cognitive restraint (CR), emotional eating (EE), and uncontrolled eating (UE). Between-group differences at each time point (0, 6 and 12 months) were evaluated with the Mann-Whitney U test. Within-group changes over time were analyzed using the Friedman test, followed by Wilcoxon signed-rank tests for pairwise comparisons.

**Results:**

Continuous self-monitors exhibited significantly higher CR at six months (p < 0.01) and lower BES across all time points (p < 0.05, < 0.01, < 0.001) when compared to non-continuous self-monitors. Within-group analysis revealed that in the continuous self-monitor group, BES, UE and EE showed significant reductions over time (p < 0.0001), while CR increased (p < 0.0001).

**Conclusion:**

Continuous self-monitoring appears to support sustained improvements in eating behaviors, including increased CR and reductions in disordered eating patterns over time. These findings highlight the potential of tailored mHBCSS, using evidence-based strategies, personalized feedback, and subtle reminders, to enhance engagement, promote healthier habits, and reduce the risk of disordered eating.

## Introduction

Behavior change support systems (BCSS) is a comprehensive term within the field of information systems, aimed at inducing cognitive and/or emotional transformation in users by facilitating the transition from their present state into another better planned state ultimately leading to behavior change.^1,2^ To achieve the desired outcomes, a variety of persuasive software features can be implemented using the persuasive systems design (PSD) framework.^3^ This framework is widely applied in the development of mobile health behavior change support systems (mHBCSS) and has been shown to be effective in driving positive health outcomes.^4^

Nonetheless, some mHBCSS adopt a ‘one-size-fits-all’ design, which can hinder the attainment of intended outcomes, as individuals are motivated by different features,^5^ and different health domains are also supported more effectively by distinct software features.^6,7^ Therefore, it is crucial to understand which features are most effective for different individuals and health domains to ensure that mHBCSS can be tailored and optimized for better impact. Among the various strategies implemented through PSD, self-monitoring has been studied and recognized as a high-evidence feature.^8^ It has emerged as a key persuasive strategy, especially valuable in weight management contexts.^9,10^ Integrating it into mHBCSS is a key approach for enhancing user engagement with health behaviors and digital interventions, thereby promoting favorable outcomes.

Previous research has shown that persuasive strategies such as tracking and monitoring are frequently applied in interventions targeting eating behaviors.^11^ These strategies are often embedded within mHBCSS to encourage engagement and support behavior change. By enhancing self-awareness and providing guidance through feedback, these PSD features may help individuals regulate their eating behaviors, including controlling emotional eating, increasing cognitive restraint, and reducing episodes of overeating, also important factors in healthy weight management. Given that these specific eating behaviors contribute directly to obesity-related outcomes, leveraging PSD features in mHBCSS may offer a targeted approach to support healthy eating patterns while mitigating risks associated with disordered eating.

Obesity and overweight are significant public health concerns, contributing to health risks such as type 2 diabetes and heart disease.^12^ In response, mHBCSS have become essential tools for weight management, supporting individuals in achieving and maintaining a healthy weight.^13^ These systems facilitate self-monitoring, which enhances self-awareness and allows users to track progress, reflect on behaviors, and make more informed and conscious decisions.^14^ A recent systematic review highlighted that daily weighing has been shown to be an effective strategy for weight loss and maintenance.^10^

Evidence has shown that PSD features, including primary task, dialogue, and credibility support, along with unobtrusive design, can enhance system's persuasiveness, promote goal setting, and ultimately influence health outcomes such as body mass index (BMI) reduction.^15^ Such findings highlight the potential for well-designed persuasive software features to influence user engagement and behavioral outcomes.

However, despite the benefits of self-monitoring, its weaknesses may include feelings of oppression and punishment, as calorie counting and perhaps even constant tracking can sometimes contribute to the development or worsening of health issues such as eating disorders,^14^ particularly when self-monitoring is used rigidly or without consideration of individual motivations.^16^ Given that adults with obesity also may engage in binge eating or exhibit probable binge eating disorder,^17,18^ characterized as a subjective loss of control over eating or eating notably more or differently than usual,^19^ it is crucial to investigate how self-monitoring influences eating behaviors and to assess its potential implications for disordered eating patterns.

This study evaluates the 12-month impact of continuous vs. non-continuous weight self-monitoring on eating behaviors (cognitive restraint, emotional eating, uncontrolled eating, and binge eating) in individuals with obesity. Findings aim to inform the design of personalized mHBCSS that promote weight management while minimizing the risk of disordered eating patterns.

## Theoretical background

### Eating behavior

Self-reported, psychometrically validated assessments play an important role in the field of eating behavior, serving not only as essential tools for research and clinical practice but also as effective means of screening individuals who may be at risk for an eating disorder diagnosis.^20^

The Binge Eating Scale (BES) is a 16-item unidimensional self-report questionnaire designed to assess the severity of binge eating, capturing both behavioral aspects (e.g., overeating) and emotional or cognitive components (e.g., feelings of guilt, fear of losing control over eating).^21^ Additionally, higher BES scores are positively linked to a tendency to engage in unrealistically restrictive dieting and to feelings of low self-efficacy in adhering to such diets.^21^

The Three-Factor Eating Questionnaire - Revised 18-item Version (TFEQ-R18)^22^ is a shorter version of the Stunkard and Messick TFEQ and comprises three different domains corresponding to cognitive restraint (CR), emotional eating (EE), and uncontrolled eating (UE).

“Restrained eating” refers to the intentional restriction of food intake for weight control.^23,24^ Stunkard and Messick^25^ later defined “cognitive eating restraint”, a mental approach to reduce energy intake for weight management, which is also commonly referred to simply as “cognitive restraint”. While associated with lower food intake and successful weight loss,^26,27^ this construct is not uniform. Excessive restraint may lead to disordered eating patterns, such as binge eating, hindering long-term weight loss. In contrast, a more flexible approach to restraint supports healthier eating behaviors and greater weight loss success.^26^

UE refers to episodes where individuals lose control over their eating. It can be triggered by extreme hunger or external cues and may be associated with a lack of regulation in eating habits, particularly in response to physiological hunger or emotional states.^22^ EE usually refers to eating in response to negative emotions, such as stress, anxiety, or loneliness. These individuals typically use food to cope with emotional distress, which can lead to overeating or binge eating.^22^ Both have been linked to negative body image.^28^

Previous research has also indicated that higher scores in CR and EE are associated with an increased BMI. Additionally, when BMI was treated as a continuous variable, a statistically significant yet weak positive correlation was observed between UE scores and BMI.^29^ Recently, a study using the TFEQ-R18 also found similar associations between BMI and UE and EE.^30^

### Persuasive systems design

Behavior change support systems aim to influence attitudes or behaviors without relying on coercion or deception. The persuasive system design (PSD) model outlines key steps in designing such systems, including understanding challenges, analyzing the context, and designing software features. The seven postulates of persuasive systems derived from socio-psychological theories, emphasize transparency, unobtrusiveness, and balancing utility with ease of use.^3^

The primary task support category of software features focuses on designing features that align with individuals’ behavioral goals, track their progress, and address key aspects such as reducing cognitive load and preventing disorientation during system use. These include self-monitoring (e.g., tracking behavior like weight or exercise), personalization, tailoring, tunneling, reduction, rehearsal, and simulation.^3^

The Computer-Human Dialogue Support category involves human-computer interaction features, such as reminders, suggestions, praise, and rewards, to engage users and guide them toward their goals. Credibility Support enhances trust by providing, for instance, expertise and authority, ensuring users can accept recommendations and may believe in desired outcomes. Finally, the Social Support category motivates users through social influence, offering, for example, recognition and enabling social comparison to encourage goal achievement.^3^

## Methods

### Sample and mHBCSS

This study comprises the intervention group of a randomized, open, wait-list controlled two-arm trial. The study was approved by the Ethics Committee of Northern Ostrobothnia Hospital District (approval number 138/2020), and the Finnish Medicines Agency, with the mHBCSS classified as an investigational medical device.

The trial aimed to evaluate the effectiveness of the mHBCSS in facilitating weight loss. The inclusion and exclusion criteria were described in previous publication.^31^

Participants were randomly assigned to one of two groups: the intervention group (n = 100), which received immediate access to the mHBCSS at the start of the trial, or the wait-list group (n = 100), who began using the application after a 6-month waiting period. Participants in both groups attended the research unit for health assessments conducted by a study nurse at baseline, 6 months, 12 months, and 18 months. Additionally, eating behavior questionnaires were completed by participants at the baseline, 6-month and 12-month marks of mHBCSS usage.

**Figure 1.**
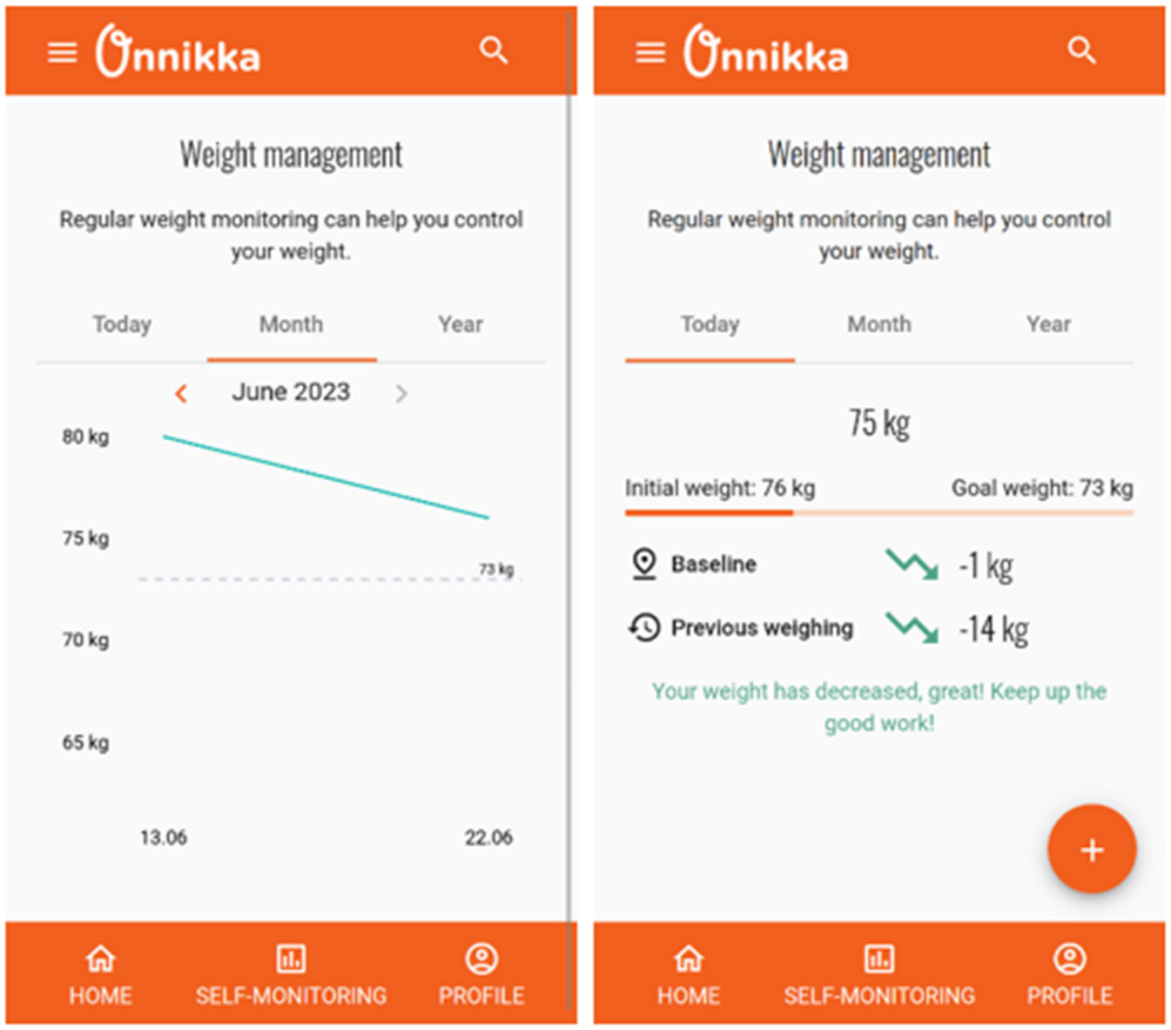
Screenshots of the weight self-monitoring feature.

The mHBCSS, named “Onnikka,” incorporated several features to support participants throughout the study. Self-monitoring features (primary task support) allowed users to track their weight, log their food and exercise activities, and maintain a mood diary. The intervention included dialogue support features, consisting of suggestions and reminders. Participants were expected to weigh themselves at least once a week, with reminders sent on Mondays. Credibility support was provided through expert-created content, links to evidence-based resources, and a transparent privacy policy. Furthermore, participants received personalized feedback and praise on their weight loss progress after recording their weight in the application (Figure 1).

Our study sample consisted of the intervention group (n = 98) after exclusion of two participants due to cortisone use and pregnancy. These two circumstances could introduce bias into the results, as cortisone use, and pregnancy affect weight.

For analysis purposes, participants were classified post-hoc into two groups based on their self-monitoring behavior regarding weight input. The “continuous self-monitors” group included individuals who continuously monitored their weight for at least 6 months, continuing through the 12-month time point when access to the mobile application was discontinued. The “non-continuous self-monitors” group consisted of participants who interrupted their weight monitoring before reaching the 6-month mark of using the mHBCSS application. Participants were considered “continuous” if they continued recording weight in the app beyond 168–209 days (average 188 days), and “non-continuous” if they stopped recording before this period. This classification was based on the actual self-input weight data logged by participants during the study.

Terms ‘sex’ and ‘gender’ are used in our research and publication as binary categorization designated at birth.

### Sample size and justification

The randomized trial used an a priori sample-size calculation for the primary outcome (change in weight): the trial was powered to detect a 3.2 kg between-group difference assuming an SD of change of 6.7 kg (69 participants per group required; target 100 per group after assuming 30% dropout, 80% power, two-sided α = 0.05).^31^ This manuscript reports a secondary analysis restricted to the intervention arm. After excluding the abovementioned two participants the available sample was n = 98 (continuous self-monitors n = 75; non-continuous self-monitors n = 23). Because this is a secondary, convenience analysis using all available intervention-arm participants, no new prospective sample-size calculation was performed. For transparency we report post-hoc detectable-effect estimates for the observed split: using a two-sample approximation (two-sided α = 0.05), the minimum detectable standardized effect (Cohen's d) is ≈ 0.67 at 80% power and ≈ 0.77 at 90% power. These values indicate the analysis is powered to detect moderate-to-large, standardized differences.

### Statistical analysis

Due to non-normality (Shapiro-Wilk test), data were analyzed using non-parametric tests. Missing data (3.18%) were imputed using the median. The Mann-Whitney U test was used for between-group comparisons, the Friedman test for within-group changes, and the Wilcoxon signed-rank test for pairwise comparisons (0 m to 6 m, 6 m to 12 m, and 0 m to 12 m). All analyses were performed in R (version 4.4.0), with a significance threshold of p < 0.05.

### Outcome variables

The BES^21^ and TFEQ-R18^22^ were used to assess eating behaviors. Both questionnaires were previously applied in the Finnish language.^29,32^

The BES consists of 16 items organized into groups of 3 or 4 statements. Respondents are asked to select the statement that best reflects their behavior. The total score ranges from 0 to 46, with higher scores indicating more severe binge eating symptoms.^21^ The BES scores were treated as a continuous variable in this analysis.

Additionally, the TFEQ-R18 was employed to assess three distinct aspects of eating behavior: CR, EE, and UE. Each item on the questionnaire is rated on a 4-point scale (definitely true/mostly true/mostly false/definitely false). Following the literature, the raw scale scores were transformed to a 0–100 scale [((raw score - lowest possible raw score)/possible raw score range)×100]. Higher scores are also indicative of greater CR, EE or UE^33^ (i.e., more conscious restriction of food intake for CR, more frequent eating in response to emotions for EE, and more difficulty controlling food intake for UE).

## Results

The analyzed dataset consisted of 12 males (12.2%) and 86 females (87.8%). Participants exhibited a mean total weight self-monitoring frequency of 28.6 times (SD 22.5), while the median was 26 (IQR 21.8). The mean of total weight self-monitoring days for females was 27.9 days (SD 21.0), with a median of 27.5 (IQR 24.8). For males, the mean was 34 days (SD 32.4), with a median of 24 (IQR 9.3). The difference in self-monitoring days between men and women was not statistically significant (U = 511, p = 0.96).

Continuous self-monitors exhibited a mean of 34.6 self-monitoring days (SD = 22.4), with a median of 30 days (IQR = 16). In contrast, the non-continuous group had a mean of 9.2 days (SD = 5.9), with a median of 8 days (IQR = 9). The difference in total self-monitoring days between the continuous and non-continuous groups was statistically significant (U = 66, p < 0.001).

The individuals’ detailed demographic information is provided in Table 1.

**Table 1.** Demographic information.

Characteristics	Frequency (n)	Percentage (%)
Sex		
Male	12	12.2
Female	86	87.8
Marital status		
Unmarried	12	12.2
Married	59	60.3
Cohabitation	16	16.3
Divorced	10	10.2
Widow	1	1
Basic education		
Primary school 1-6^a^	0	0
Primary school 1–9^b^	23	23.5
Primary School 4–9^a^	8	8.2
High school graduate/ secondary school graduate	67	68.3
Professional training		
Professional course (at least 4 months), earlier employment course in Finnish	1	1
Other employment course (at least 4 months)	1	1
Vocational school	15	15.3
University of applied sciences	46	47
University	24	24.5
None of the above	11	11.2

Notes: n = 98, ^a^old education system in Finland ^b^current education system in Finland.

The first analysis compared continuous and non-continuous self-monitors at 0, 6, and 12 months (Table 2). Significant differences were found for CR, with continuous self-monitors showing higher scores at 6 months (Median [IQR]: 55.6 [22.3] vs. 44.4 [11.2]; U = 529.5, p < 0.01). Continuous self-monitors also had lower BES scores at all time points (0m: Median [IQR] = 11^8–16^ vs. 14 [12–17.5]; U = 1114, p < 0.05; 6m: 7 [4.5–10] vs. 12 [8–16.5]; U = 1235.5, p < 0.01,; 12m: 6 [4–9.5] vs. 12^7–16^; U = 1284.5, p < 0.001). No significant differences were observed between groups for EE, UE, or baseline/12-month CR (all p > 0.05).

**Table 2.** Differences in eating behaviors between continuous and non-continuous weight self-monitors.

Variable	Time point	Continuous Median (IQR)	Non-continuous Median (IQR)	U Statistic	P-value	r_rb (Effect size)
Cognitive Restraint	0m	38.9 (19.5)	38.9 (8.3)	846.5	0.895	0.02
	6m	55.6 (22.3)	44.4 (11.2)	529.5	*<0*.*01*	*0*.*39*
	12m	50 (5.6)	50 (8.4)	837	0.830	0.03
Emotional Eating	0m	44.4 (33.4)	44.4 (39)	843.5	0.875	0.02
	6m	33.3 (22.2)	33.3 (28)	1078	0.066	−0.25
	12m	33.3 (33.4)	44.4 (11.1)	1036	0.137	−0.20
Uncontrolled Eating	0m	40.7 (22.3)	48.1 (29.7)	985	0.306	−0.14
	6m	33.3 (20.4)	37 (29.7)	1036	0.146	−0.20
	12m	29.6 (33.4)	37 (19.5)	1080.5	0.068	−0.25
Binge Eating	0m	11 (8–16)	14 (12–17.5)	1114	*<0*.*05*	*-0*.*29*
	6m	7 (4.5–10)	12 (8–16.5)	1235.5	*<0*.*01*	*-0*.*43*
	12m	6 (4–9.5)	12 (7–16)	1284.5	*<0*.*001*	*-0*.*49*

Note: After median imputation,^34,35^ the Mann-Whitney U tests were performed to compare the “continuous” vs “non-continuous” self-monitoring groups across different time points (0, 6, and 12 months) for various measures (Cognitive Restraint, Emotional Eating, Uncontrolled Eating, Binge Eating). The p-values and U statistics are presented for each comparison. Significant p-values are reported as less than the threshold value when they are smaller than the specified cutoff (p < .05, p < .01, p < .001). IQR represents the interquartile range. R_rb: rank-biserial correlation.

Effect sizes for group differences were estimated using rank-biserial correlations (r_rb), which indicate both the magnitude and direction of the effect. Positive values reflect higher scores in continuous self-monitors, whereas negative values indicate higher scores in non-continuous self-monitors. For CR, continuous self-monitors had higher scores at 6 months (r_rb = 0.39), indicating a moderate-to-large increase compared with non-continuous self-monitors. EE and UE showed small-to-moderate negative effects at later time points (EE 6m: r_rb = -0.25; EE 12m: r_rb = -0.20; UE 12m: r_rb = -0.25), reflecting slightly higher scores in non-continuous self-monitors. BES was consistently lower in continuous self-monitors, with moderate-to-large negative effects at 6 and 12 months (r_rb = -0.43 and −0.49), indicating clinically meaningful reductions in binge eating associated with continued self-monitoring.

After adjusting for baseline values using rank ANCOVA (Table 3), continuous self-monitors showed significantly higher CR scores at 6 months compared with non-continuous self-monitors (adjusted difference = 14.18, 95% CI: 2.58–25.77, p = 0.017). EE and BES were significantly lower in continuous self-monitors (EE: −16.16, 95% CI: −26.34 to −5.99, p = 0.002; BES: −16.11, 95% CI: −27.01 to −5.22, p = 0.004). The difference in UE did not reach statistical significance (adjusted difference = –8.44, 95% CI: −18.47 to 1.59, p = 0.098). At 12 months, continuous self-monitors had significantly lower EE (adjusted difference = -15.13, 95% CI: −25.18 to −5.08, p = 0.004), UE (adjusted difference = -12.43, 95% CI: −22.58 to −2.28, p = 0.017), and BES (adjusted difference = -19.53, 95% CI: −29.72 to −9.34, p < 0.001) compared with non-continuous self-monitors. CR scores were not significantly different between groups at 12 months (adjusted difference = –5.81, 95% CI: −18.94 to 7.32, p = 0.382).

**Table 3.** Adjusted between-group differences in eating behaviors at 6 and 12 months (rank ANCOVA).

Variable	6 Mo adjusted effect (95% CI)	P-Value	12 Mo adjusted effect (95% CI)	P-Value
Cognitive Restraint	14.18 (2.58, 25.77)	p < 0.05	−5.81 (−18.94, 7.32)	p = 0.382
Emotional Eating	−16.16 (−26.34, −5.99)	p < 0.01	−15.13 (−25.18, −5.08)	p < 0.01
Uncontrolled Eating	−8.44 (−18.47, 1.59)	p = 0.098	−12.43 (−22.58, −2.28)	p < 0.05
Binge Eating	−16.11 (−27.01, −5.22)	p < 0.01	−19.53 (−29.72, −9.34)	p < 0.001

Note: The adjusted group effect represents the difference between continuous vs. non-continuous self-monitors at 6 and 12 months, adjusted for baseline ranks. Significant p-values are reported as less than the threshold value when they are smaller than the specified cutoff (p < 0.05, p < 0.01, p < 0.0001).

These results suggest that sustained adherence to weight self-monitoring is associated with long-term improvements in emotional and binge-related eating behaviors, while the effect on CR may diminish over time.

The second analysis assessed within-group changes over time using the Friedman test (Table 4). Continuous self-monitors showed significant BES changes, with Wilcoxon tests revealing differences between 0–6 and 0–12 months. No significant changes were found for non-continuous self-monitors, so Wilcoxon tests were not conducted.

**Table 4.** Comparison of each eating behavior across time points within groups.

Statistical Test						Eating Behavior							
		Cognitive Restraint		Emotional Eating				Uncontrolled Eating		Binge Eating			
		Test	p-value	Effect size	Test	p-value	Effect size	Test	p-value	Effect size	Test	p-value	Effect size
**Friedman Test** (Continuous)		χ²(2) = 28.8	*<0.0001*	Kendall's W = 0.58	χ²(2) = 23.1	*<0.0001*	Kendall's W = 0.75	χ²(2) = 54.6	*<0.0001*	Kendall's W = 0.81	χ²(2) = 53.3	*<0.0001*	Kendall's W = 0.76
**Wilcoxon Signed-Rank** (Continuous)	0 m to 6m	V = 356	*<0*.*0001*	r = 0.57	V = 1540	*<0.0001*	r = 0.57	V = 2112.5	*<0.0001*	r = 0.62	V = 2122.5	*<0.0001*	r = 0.63
	6 m to 12m	V = 1371.5	0.05	r = 0.23	V = 561.5	0.61	r = 0.06	V = 1370	0.15	r = 0.17	V = 1279	0.27	r = 0.13
	0 m to 12m	V = 583.5	*<0*.*001*	r = 0.42	V = 1140.5	*<0.0001*	r = 0.48	V = 2012.5	*<0.0001*	r = 0.67	V = 2248	*<0.001*	r = 0.68
**Friedman Test** (Non-Continuous)		χ²(2) = 8.07	*<0.05*	Kendall's W = 0.38	χ²(2) = 1	0.61	Kendall's W = 0.76	χ²(2) = 4.17	0.13	Kendall's W = 0.77	χ²(2) = 3.43	0.18	Kendall's W = 0.67
**Wilcoxon Signed-Rank** (Non-Continuous)	0 m to 6m	V = 45	0.05	r = 0.42	NA	NA		NA	NA		NA	NA	
	6 m to 12m	V = 72.5	0.14	r = 0.31	NA	NA		NA	NA		NA	NA	
	0 m to 12m	V = 37	*<0*.*05*	r = 0.53	NA	NA		NA	NA		NA	NA	

Note: χ²(df) represents the Chi-squared statistic with degrees of freedom (df) for the Friedman test. V refers to the test statistic from the Wilcoxon signed-rank test for pairwise comparisons. NA: not applicable. Significant p-values are reported as less than the threshold value when they are smaller than the specified cutoff (p < 0.05, p < 0.01, p < 0.0001).

Effect sizes were calculated for both Friedman and Wilcoxon tests to quantify the magnitude of changes over time. For participants who continued monitoring (continuous group), Kendall's W indicated moderate to large effects for all eating behaviors (CR: 0.58; EE: 0.75; UE: 0.81; BES: 0.76). Pairwise comparisons using the Wilcoxon signed-rank test showed small to large effects, with the largest observed for BES (r = 0.68, 0→12 months) and UE (r = 0.67, 0→12 months). In the non-continuous group, significant changes were observed only for CR, with moderate effect sizes across all time intervals (r = 0.42–0.53). Effect sizes for EE, UE, and BES in the non-continuous group were not calculated because the overall Friedman tests were non-significant. These findings suggest that continued monitoring is associated with stronger and more consistent changes in eating behavior over time.

The Friedman test also revealed significant time-point differences in UE and EE for continuous self-monitors (UE: χ²(2) = 54.6, p < 0.0001, Kendall's W = 0.81; EE: χ²(2) = 23.1, p < 0.0001, W = 0.75), with significant changes between 0–6 months (UE: V = 2112.5, p < 0.0001, r = 0.62; EE: V = 1540, p < 0.0001, r = 0.57) and 0–12 months (UE: V = 2012.5, p < 0.0001, r = 0.67; EE: V = 1140.5, p < 0.0001, r = 0.48). No significant changes were found for non-continuous self-monitors. Regarding CR, significant time-point differences were found for both groups. Continuous self-monitors showed significant changes between 0–6(V = 356, p < 0.0001, r = 0.57) and 0–12 months (V = 583.5, p < 0.001, r = 0.42), though the 6–12 month difference (V = 1371.5, p = 0.05034, r = 0.23) was marginally above the threshold for significance. Non-continuous self-monitors showed significant changes only between 0–12 months (V = 37, p < 0.05, r = 0.53), with the 0–6 month difference being marginally under significance (V = 45, p = 0.04527, r = 0.42). A graphical representation is provided in Supplemental File 1.

Finally, there were no significant differences in education or professional training between continuous and non-continuous self-monitors. Fisher's exact tests confirmed that the distribution of both basic education (Fisher's exact p = 0.393) and professional training (Fisher's exact p = 0.210) did not differ between groups. These results suggest that adherence to self-monitoring was not associated with participants’ educational background.

## Discussion

This study examined the impact of weight self-monitoring on eating behavior over 12 months using a mHBCSS. Our findings suggest that continuous self-monitoring was associated with greater improvements in CR and binge eating tendencies compared to non-continuous self-monitoring. Specifically, continuous self-monitors showed significantly higher CR at 6 months and lower BES at all time points. Within the continuous self-monitor group, BES, UE, and EE decreased significantly over time. CR also increased significantly from baseline to 6 months and from baseline to 12 months, while the change from 6 to 12 months was only marginal, suggesting that some initial gains in dietary control were maintained over the longer term. Despite this, continuous self-monitoring was associated with overall improvements in eating behaviors and dietary control over 12 months, highlighting its potential value as part of a digital intervention for weight management in individuals with obesity.

Self-monitoring is not an isolated tool but a core feature of health informatics, supporting behavior change by tracking progress, and enabling to provide feedback and enhance awareness of one's own behavior. Moreover, to maximize its effectiveness, it should be integrated with other mHBCSS components, such as Computer-Human Dialogue and Credibility Support features.

Recent research evaluating the same mHBCSS investigated in this study has shown that the relationship between Dialogue Support and Credibility Support with Primary Task Support is significant.^15^ For a system to achieve meaningful outcomes, it must not only facilitate self-monitoring but also provide reliable information and effective communication with users. Furthermore, Dialogue Support features should be implemented in an unobtrusive manner to positively impact perceived persuasiveness and reduce BMI.^15^ Unobtrusive design, for example, may include passive data collection (e.g., connected wearable device that automatically records step count or heart rate), use of intelligent reminders that adjust to user behavior, and quick entry options for frequent activities.

Practically, in our intervention, Dialogue Support and Credibility Support were implemented through specific features of the mHBCSS. Dialogue Support included scheduled reminders and suggestions, which were delivered unobtrusively to encourage consistent self-monitoring without overwhelming users. Credibility Support involved providing reliable, evidence-based information and clear guidance, which likely enhanced participants’ trust in the system and adherence to its recommendations. These features may have contributed to the observed improvements in eating behaviors as well. For example, continuous self-monitors who received more frequent reminders and suggestions may have shown larger reductions in BES and UE, suggesting that the integration of these main software feature categories reinforced engagement and supported healthier eating patterns. By linking these features to the outcomes, we illustrate how specific components of the intervention, beyond self-monitoring alone, may have facilitated behavioral change.

Striking a balance between useful, credible information and unobtrusiveness is crucial for sustaining self-monitoring behavior and the overall efficacy of the mHBCSS. Self-monitoring's true potential is realized when combined with complementary features that support, guide, and motivate users. Integrating self-monitoring into daily routines, such as through voice-based assistants, and providing summarized feedback rather than constant prompts, could help maintain engagement. Simple interfaces and user control over detailed information may be capable to reduce cognitive load and prevent overwhelm, enhancing long-term adherence and positive behavioral outcomes.

Self-monitoring not only may support behavior change but also can empower individuals to feel more in control of managing their health.^36^ However, as Fiske, Buyx and Prainsack^37^ point out, this empowerment should align with professional guidance to ensure safety and effectiveness. They emphasize that digital self-care should not replace medical expertise, as “there has to be a doctor-steered process,” [37(p.4)] with personal relationships remaining central to the meaningful use of new information technologies. Thus, they argue that while self-monitoring promotes autonomy, it should complement, not substitute, professional healthcare.

Previous research suggests that self-monitoring behavior could lead to eating disorder patterns^38^ or that diet and fitness apps could exacerbate eating disorder behaviors due to the quantified self-movement and also due to visual cues and feedback.^39^ However, our findings provide further evidence that, when used correctly (e.g., delivered at appropriate times, limited in frequency to avoid overwhelming users, and respecting user autonomy through reduced and unobtrusive reminders), self-monitoring does not contribute to disordered eating behaviors but rather helps encourage healthier eating patterns over time. Their results may differ from ours due to variations in the type of self-monitoring, such as calorie tracking,^38^ and differences in research samples, including a group of 24 women (ages 18–23) in a qualitative study.^39^ Additionally, Eikey^39^ identified challenges in fitness apps, such as the “overabundance of quantification” and “visual cues do not always match users’ goals”. These challenges highlight the potential harms that can arise when the PSD framework is not systematically applied. To mitigate the negative effects of excessive quantification, features like reduction and personalized suggestions to users’ goals should be considered, both of which pertain to the Primary Task Support category.

Our findings also agree with Jospe et al.,^40^ who found that self-monitoring, including the use of diet apps and self-weighing, did not have adverse effects on disordered eating behaviors in adults attempting to lose weight. In their study, there were no significant differences between groups in terms of key disordered eating behaviors, such as binge eating, self-induced vomiting, or laxative misuse. Similarly, our research demonstrates that continuous self-monitoring is not associated with the development of disordered eating behaviors but rather appears to support healthier eating patterns over time.

The lack of significant improvements in the non-continuous weight self-monitoring group (for BES, UE and EE, Table 4) emphasizes the importance of consistency. A potential barrier that individuals may face in maintaining continuous self-monitoring could be related to the perception that it is tedious, which may discourage long-term engagement.^14^ Intrinsic factors such as time constraints, lack of motivation, or competing priorities may also impede sustained self-monitoring. To overcome these barriers and make self-monitoring more sustainable in real-world situations, strategies could include simplifying the process by incorporating more automated or passive tracking features, offering personalized reminders and motivational support, and integrating self-monitoring into daily routines to reduce the perceived burden. Additionally, gamification elements or rewards for frequent tracking perhaps could enhance motivation and engagement.

Interestingly, within the non-continuous self-monitoring group, CR showed a slight but gradual increase over time. The significant improvement from baseline to twelve months suggests that some degree of dietary restraint was developed, possibly due to increased awareness of eating behaviors from initial mHBCSS use, sporadic engagement with self-monitoring, since these participants were primarily active in the early stages of the intervention, or due to other intervention-related features.

Regarding the study limitations, we acknowledge the use of self-reported scales, which may be subject to biases such as recall bias. Furthermore, the study sample was predominantly composed of females and most of participants also had a professional healthcare background, which may limit the generalizability of the findings to other populations. However, this sample composition is frequent with the findings of a recent systematic review by Hallock, Ufholz, and Patel,^10^ which examined the latest evidence on self-monitoring and its impact on weight loss and weight-related behaviors. Their review highlighted that most studies in this domain predominantly include white women, indicating that our sample aligns with the broader trends in this area of research. Future studies could consider investigating other factors that might influence effectiveness of self-monitoring, such as individual differences (e.g., psychological traits) or comparing the type of self-monitoring method used (e.g., food tracking, exercise tracking).

We also acknowledge that the comparison between continuous and non-continuous self-monitors is post-hoc, as the original study was not designed to examine these subgroups. Consequently, we cannot definitively attribute observed differences in eating behaviors solely to adherence to weight self-monitoring, since other components of the intervention (e.g., reminders and suggestions) or unmeasured participant characteristics may have contributed. Finally, although we observed associations between self-monitoring adherence and eating behavior outcomes, further research is needed to examine individual differences, psychological traits, and alternative self-monitoring methods (e.g., food tracking, exercise tracking) that may influence intervention effectiveness.

## Conclusion

Our study indicates that continuous weight self-monitoring within this intervention was associated with healthier eating behaviors over 12 months, without evidence of increased eating disorder risk in this population. The 12-month longitudinal analysis reinforces the need for mobile health apps that are not only effective but also tailored to users’ unique needs and preferences. To maintain sustained engagement, these apps must integrate evidence-based principles, personalized feedback, and non-disturbing reminders. When personalized and unobtrusive, weight self-monitoring can encourage healthier eating patterns and contribute to positive long-term healthy eating behaviors without increasing the risk of eating disorders.

## Supplemental Material

sj-docx-1-dhj-10.1177_20552076251395530 - Supplemental material for Mobile weight self-monitoring adherence and eating behavior changes: A secondary analysis of a 12-month RCT Supplemental material, sj-docx-1-dhj-10.1177_20552076251395530 for Mobile weight self-monitoring adherence and eating behavior changes: A secondary analysis of a 12-month RCT by Renata Savian Colvero de Oliveira, Sharon Nabwire, Silja Rantaiso, Heta Merikallio, Markku J Savolainen, Janne Hukkanen and Harri Oinas-Kukkonen in DIGITAL HEALTH
