# Supplementary material for: Mobile weight self-monitoring adherence and eating behavior changes: A secondary analysis of a 12-month RCT
Source: Digit Health. 2025 Dec 10;11:20552076251395530. doi: 10.1177/20552076251395530 (PMC12696281; doi:10.1177/20552076251395530)
Supplement: sj-docx-1-dhj-10.1177_20552076251395530 - Supplemental material for Mobile weight self-monitoring adherence and eating behavior changes: A secondary analysis of a 12-month RCT [file sj-docx-1-dhj-10.1177_20552076251395530.docx]

**Supplemental File 1. Significant changes in BES, CR, EE and UE over time**

| 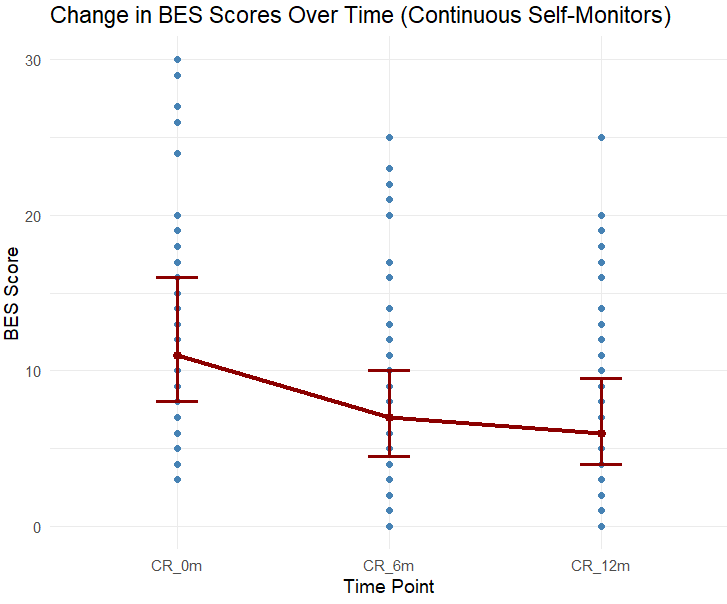 |
| --- |

| 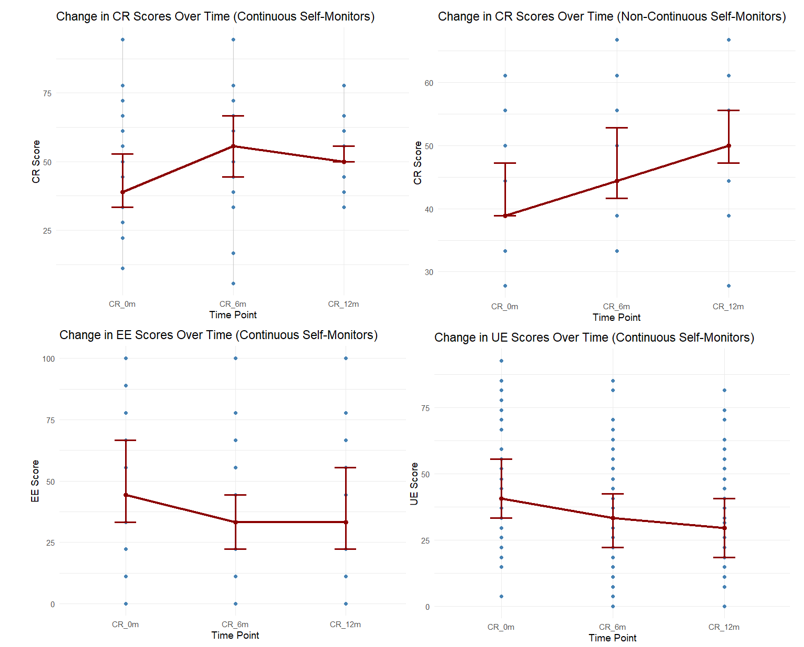 |
| --- |

Note: Significant Differences Found in Friedman Test Within Groups. BES: Binge Eating Scale, CR: Cognitive Restraint, EE: Emotional Eating, UE: Uncontrolled Eating. The red line connects median scores at each time point. Error bars represent the interquartile range.
